# Visualization of FDA Adverse Drug Reaction Reports: Development and Usability Study of the VisDrugs Web Server

**DOI:** 10.2196/71519

**Published:** 2025-07-31

**Authors:** Renjun Yang, Nuoya Yin, Yang Zhang, Francesco Faiola

**Affiliations:** 1 State Key Laboratory of Environmental Chemistry and Ecotoxicology, Research Center for Eco-Environmental Sciences, Chinese Academy of Sciences, 18 Shuangqing Road, Haidian District, Beijing, 100085, China, 86 01062849096; 2 College of Resources and Environment, University of Chinese Academy of Sciences, Beijing, China; 3 Department of Pharmacy, Beijing Friendship Hospital, Capital Medical University, Beijing, China

**Keywords:** FDA Adverse Event Reporting System, adverse drug reactions, drug safety, data visualization, website, FAERS, ADR, Food and Drug Administration

## Abstract

**Background:**

Adverse drug reactions (ADRs) are a major concern in drug safety, and the FDA Adverse Event Reporting System (FAERS) provides valuable ADR data. However, analyzing FAERS data is complex and requires bioinformatics expertise. Despite the vast amount of ADR data available, there is a lack of user-friendly tools that enable efficient visualization and comparison of ADRs for researchers and health care professionals.

**Objective:**

This study aimed to develop VisDrugs, a web-based platform that simplifies ADR visualization and comparison using FAERS data. The platform was designed to assist researchers and clinicians in assessing drug safety through interactive and interpretable graphical representations of ADR patterns.

**Methods:**

FAERS data were extracted in the American Standard Code for Information Interchange (ASCII) format, covering the period from Q3 (third quarter) 2014 to Q3 2024. About 2,700,000 reports from health care professionals, where only a single drug was implicated, were aggregated and processed using R for statistical analysis and visualization. The results are presented on a web-based platform for web-based analysis. The platform generates pie charts to visualize the most frequently reported ADRs, which are represented and analyzed using preferred terms based on the Medical Dictionary for Regulatory Activities (MedDRA) and forest plots illustrating reporting odds ratios (RORs) for these ADRs.

**Results:**

Using Paxlovid (COVID-19 treatment) and hydroxychloroquine (anti-malaria drug) as case studies, we benchmarked VisDrugs using reports for Paxlovid (n=16,708) and hydroxychloroquine (n=6150). Paxlovid was most frequently associated with “COVID-19” (ROR=47.26, 95% CI 45.22‐49.40) and “dysgeusia” (ROR=59.65, 95% CI 55.56‐64.03). Hydroxychloroquine showed strong associations with “retinal toxicity” (ROR=738.48, 95% CI 583.45‐934.71), “retinopathy” (ROR=412.27, 95% CI 344.73‐493.03), and “cardiotoxicity” (ROR=48.36, 95% CI 38.86‐60.19). In subgroup analyses, female patients had significantly higher risks of retinopathy (3.24-fold) and cardiomyopathy (13.82-fold) compared to male patients, while patients aged >50 years had higher risks of retinopathy (4.20-fold) and cardiomyopathy (7.84-fold) compared to those ≤50 years. All differences were statistically significant (*z* test, *P*<.01). The majority of findings align with existing research, thereby validating the platform’s utility. Clinical personnel have evaluated and refined the platform based on user feedback, confirming its efficacy in visualizing complex ADR data and identifying adverse effects across various drug subgroups.

**Conclusions:**

VisDrugs is a valuable tool for ADR analysis, offering an intuitive interface for exploring FAERS data. By visualizing and comparing ADRs, it helps researchers and health care providers assess drug safety efficiently. The platform’s demographic analysis features add insights into ADR variations by age and gender, supporting drug safety research. In the future, the website will include more subgroup or condition filtering options, offering personalized ADR analysis and comparison features to meet the diverse research needs of users.

## Introduction

Adverse drug reactions (ADRs) are unintended responses that are detected in patients after the use of drugs for the prophylaxis, diagnosis, or treatment of a disease at doses normally used [1]. ADRs represent an important public health challenge, contributing to significant hospital admissions and deaths worldwide [2,3]. The US Food and Drug Administration (FDA) Adverse Event Reporting System (FAERS) provides a critical database that captures reports of adverse events linked to pharmaceutical products [4,5]. With millions of reports, FAERS serves as an invaluable resource for evaluating drug safety and post-market surveillance [6-12]. FAERS is continuously updated and includes data from a wide range of sources, including health care professionals, patients, and drug manufacturers [13]. Each report in the FAERS database provides information on a specific adverse event associated with a drug, including ADR description, the drug involved, the patient’s age and sex, and other information.

Despite their vast potential for drug safety analysis, FAERS data present several challenges. The large record number poses a significant hurdle to traditional analysis methods. Moreover, the data are often unstructured and contain a considerable amount of noise, requiring sophisticated data-cleaning techniques [14]. For researchers to interpret these data effectively, a solid foundation in bioinformatics and statistical modeling is often required. The need for simplified, accessible tools for ADR analysis has become increasingly apparent. Although several web-based user interfaces, such as OpenVigil FDA and VigiAccess, facilitate access to FAERS data, conducting a detailed comparison of ADRs still necessitates performing calculations on the obtained data using bioinformatics methods [15-18]. Currently, there is no widely available web platform that offers an easy, interactive way to analyze FAERS data, especially for comparing the adverse effects of different drugs in a straightforward manner. This gap creates a barrier for researchers, clinicians, and policy makers who need quick insights into drug safety but lack the technical expertise to handle large-scale pharmacovigilance data.

In response to this challenge, we have developed the VisDrugs website, an interactive platform designed to simplify the process of analyzing drug-related adverse effects using FAERS data. The VisDrugs platform includes a comparison feature that allows users to input 2 groups of drugs and analyze the differences in their safety profiles with respect to specific adverse events. Furthermore, it conducts analyses by subgroups of age and gender to evaluate the effects of these variables on specific reactions. Visdrugs addresses a critical gap in the current landscape of drug safety analysis tools. By leveraging the extensive resources of the FAERS database and presenting them through a user-friendly interface, it enables a broader audience to engage with drug safety data.

## Methods

### Website Framework

The VisDrugs website is designed to provide 1-click analysis of FDA ADR data [19]. Users can easily use its features through any web browser, making it simple and convenient to use. The FDA ADR dataset on the site is updated annually in alignment with the latest updates from the FDA FAERS database.

VisDrugs operates on the Tencent Cloud server platform, featuring a CentOS 7.6 64-bit system with an 8-core CPU, 32 GB cache memory, 320 GB disk storage, and 22 Mbps bandwidth, supporting simultaneous web-based analyses by many researchers at the same time.

VisDrugs uses Java (version 8), Spring Boot, and MyBatis Plus to provide server-side API support. The visual frontend of the website is created using JavaScript, Vue (version 3), and Element Plus. Apache (version 9.0) is used as the web server, and nginx is used for load balancing. The MySQL database is used for backend storage of sequences, taxonomy, and user data.

The core 1-click analysis feature is powered by R language, which is a good choice for statistical analysis. The system minimizes redundant information, such as duplicated and incomplete reports, during data preprocessing, enhancing data analysis efficiency. VisDrugs provides researchers with a robust and efficient platform for FDA ADR data exploration and analysis.

### Data Retrieving, Preprocessing, and Visualization

**Figure 1.**
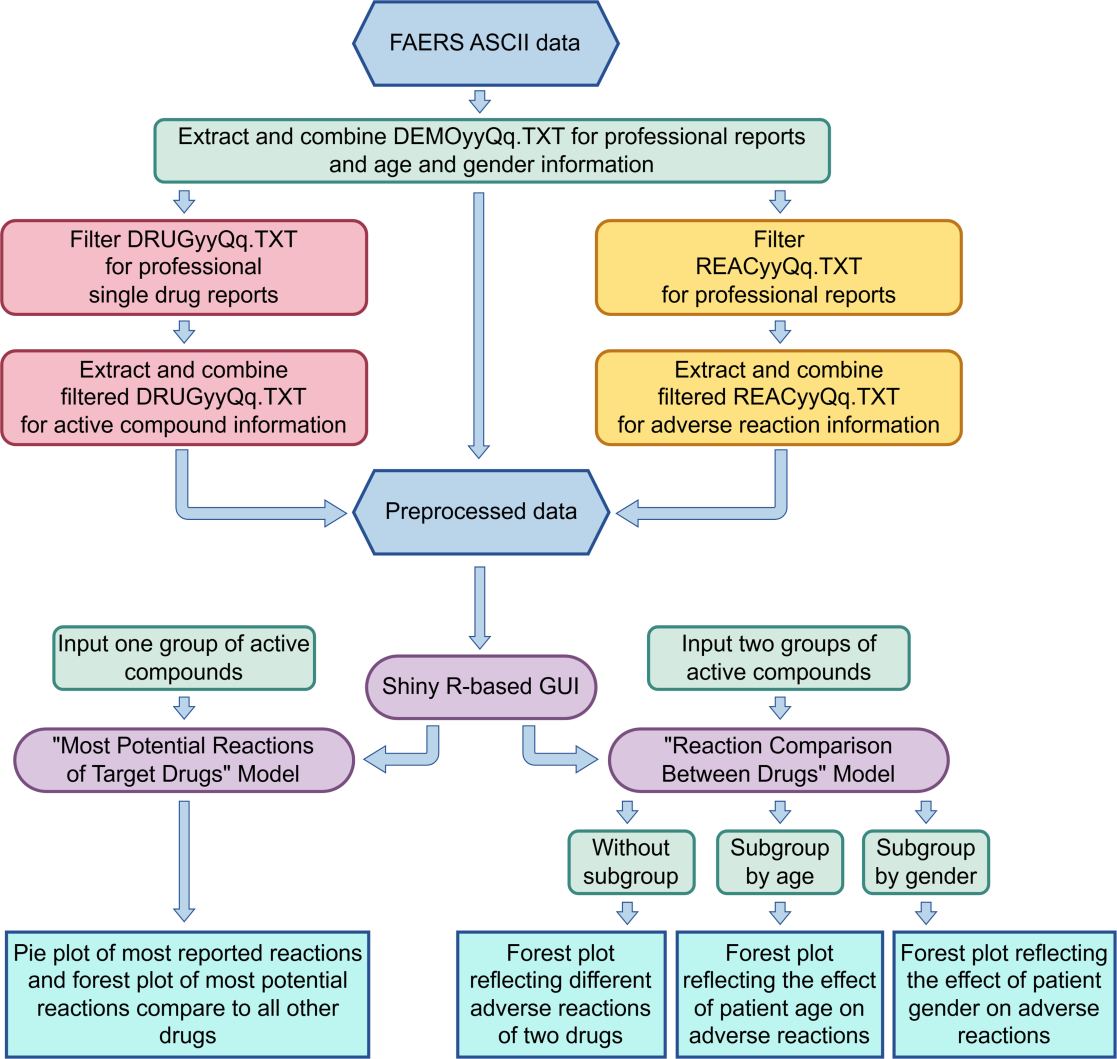
Data preprocessing flow and interactive interface operation flowchart for the VisDrugs website. ASCII: American Standard Code for Information Interchange; FAERS: Food and Drug Administration Adverse Event Reporting System; GUI: graphical user interface.

Data retrieval, preprocessing, and visualization were performed, as depicted in Figure 1. In detail, ADR data were extracted in ASCII format from the FDA database, covering the period from Q3 (third quarter) 2014 to Q1 2024. Data prior to Q3 2014 were not included mainly because they were from a time when the “DRUGyyQq.TXT” file did not contain active ingredient information for the drugs, and the data were less standardized. Starting from Q3 2014, the inclusion of drug active ingredient information and the improved data structure allowed for more accurate visualization of the results.

The data preprocessing step primarily used 3 key files: the DEMOyyQq.TXT, which contains basic case information; the REACyyQq.TXT, which includes ADR details for the patients; and the DRUGyyQq.TXT, which holds information on drug active ingredients (“Product active ingredient”) and the reported role of the drug in the adverse event (“Code for drug’s reported role in event”).

For the DEMOyyQq.TXT file, we filtered data over the past decade. Specifically, we selected data reported by 4 types of health care professionals (HP, health professional [HP]; physician [MD]; other health professional [OT]; and pharmacist [PH]) based on the abbreviation for the reporter’s type of occupation. We then aggregated quarterly data over the years to create a comprehensive DEMOyyQq.TXT dataset covering 2014‐2024. This dataset includes case ID, patient gender and age, and the occupation type of the reporter, which can be used for further interactive analysis features on the website. Additionally, we summarized the case data to filter relevant reports from REACyyQq.TXT and DRUGyyQq.TXT files.

Next, we aggregated the cases from REACyyQq.TXT reported by professionals, with the ADRs defined using preferred term (PT)–level medical terminology describing the event, using the Medical Dictionary for Regulatory Activities (MedDRA). For the DRUGyyQq.TXT file, we focused on the cases reported by professionals, extracting the active ingredients that were identified as the primary suspect drug of each report. For the aggregate files, duplicates were removed by keeping only the latest version of each case (based on the FDA’s primary ID and version number). Records missing essential information, such as the drug’s active substance name or ADR term, were excluded.

During visualization, the R language was used to analyze the preprocessed datasets, performing statistical calculations and generating graphs to display potential ADRs. In simpler terms, our self-developed ShinyR program performs statistical analysis on the aggregated preprocessed data and creates pie charts based on the total number of ADRs. Additionally, for drug ADR analysis, we use the commonly used method of reporting odds ratio (ROR) [20] to generate forest plots.

Standard MedDRA PTs were used to calculate potential ADRs, and active substances, which are standard chemical names, were applied as drug names to ensure consistency. The database will be updated yearly to incorporate the latest FAERS releases.

### Calculation of Reporting Odds Ratio and Statistical Significance Analysis

The ROR for a selected drug compared to all other drugs was calculated as reported by Rothman et al [21]:


$$ROR_{j}=\frac{N_{A,j}/N_{A,\neg j}}{N_{\neg A,j}/N_{\neg A,\neg j}}$$

where RORj is the ROR for ADRj associated with the selected drug A. N_A,j_ is the number of reports where drug A is associated with ADRj. NA,¬j is the number of reports where drug A is reported but without ADRj. N¬A,j is the number of reports where ADRj is reported but drug A is not involved. N¬A,¬j is the number of reports where neither drug A nor ADRj is reported.

The standard error of the log-transformed ROR is given by:


$$SE\left(lnROR_{j}\right)=\sqrt{\frac{1}{N_{A,j}}+\frac{1}{N_{A,\neg j}}+\frac{1}{N_{\neg A,j}}+\frac{1}{N_{\neg A,\neg j}}}$$

On the log scale, the CI is given by:


lnRORj±1.96×SE(lnRORj)

To obtain the final CI for ROR, the following equation is used:


$$CI_{ROR}=\left(e^{lnROR_{j}-1.96\times SE\left(lnROR_{j}\right)},e^{lnROR_{j}+1.96\times SE\left(lnROR_{j}\right)}\right)$$

In the forest plot, an ROR value was considered statistically significant if the lower bound of its log2-transformed 95% CI was greater than zero. To evaluate whether the RORs differed significantly between 2 subgroups (eg, age or sex), we applied a *z* test based on the log-transformed RORs and their standard errors.

The z statistic was calculated as:


$$z=\frac{ln({ROR}_{1})-ln({ROR}_{2})}{\sqrt{{SE}_{1}^{2}+{SE}_{2}^{2}}}$$

The resulting 2-sided *P* value for the *z* test was computed as:


p=2×(1−Φ(|z|))

where Φ denotes the cumulative distribution function of the standard normal distribution. A difference of at least 1.5-fold in ROR values between the 2 subgroups, combined with a *P* value<.05, was considered statistically significant.

To ensure comprehensive reporting and transparency, a detailed checklist following the Software/Tools Papers guidelines is provided (see Checklist 1).

### Ethical Considerations

This study involved secondary analysis of publicly available, deidentified data from the US FDA FAERS. No identifiable personal information was accessed or used. As the dataset is anonymized and contains no patient-level identifiers, this study was exempt from institutional review board approval in accordance with local regulations and ethical guidelines. The analysis complied with all applicable data use policies and ethical standards for research involving publicly available datasets.

## Results

### Analysis for Most Potential Reactions of Target Drugs

The VisDrugs website offers 2 selectable methods for analyzing ADRs. In the “Most Potential Reactions of Target Drugs” mode, users only need to perform 2 actions on the graphical user interface to view the ADRs most relevant to drug-of-interest’s usage: (1) type to search for the active ingredient name of the drug-of-interest. The relevant active ingredient will appear in the search selection box, where users can choose either a single active ingredient or multiple active ingredients as a whole for analysis and (2) name the target drug group and click “submit.”

After submitting, the webpage will generate two charts: (1) a pie chart showing the top 15 ADRs of the selected drug and (2) a forest plot comparing the top 15 ADRs from the pie chart that shows whether they are more likely caused by the selected drug or other drugs. Disproportionality analysis, which estimates the ROR, is used in the forest plot for detecting spontaneous signals, establishing the statistical link between drug exposure and a particular ADR [20]. The ROR, calculated using the case or non-case method, represents the odds ratio of a specific ADR relative to all other ADRs for each drug [21].

Additionally, four download buttons will be provided for users to download images or raw data: (1) high-definition pie chart of the drug’s ADRs; (2) high-definition forest plot of the drug’s ADRs; (3) an Excel table of drug ADR frequency statistics (all reactions); and (4) an Excel table of the raw data for the forest plot.

It is important to note that when typing to search for a drug, users should enter the active ingredient name of the drug. Active ingredients are more standardized than drug names and help prevent incorrect matches. Users must also select the relevant active ingredient from the list, as only selected drugs indicate that the site includes related data for that drug. Furthermore, the website provides a user guide where users can click to view detailed instructions on what information should be entered in each section and how to interpret the results.

Overall, the “Most Potential Reactions of Target Drugs” analysis function on the website provides a highly convenient method for analyzing the most common ADRs of drugs.

### Application Examples: Analyzing the Most Commonly Reported ADRs of Paxlovid and Hydroxychloroquine

We use Paxlovid, a special antiviral drug for COVID-19, and the antimalarial drug hydroxychloroquine, which was initially considered to offer therapeutic benefits in COVID-19 patients [22], but withdrew from use in COVID-19 treatment by FDA in June 2020 [23], as examples to demonstrate the “Most Potential Reactions of Target Drugs” feature on the website. Paxlovid contains 2 active ingredients: Nirmatrelvir and Ritonavir [24-26]. In this example, given the focus on Paxlovid, the analysis was conducted using the active ingredients “NIRMATRELVIR/RITONAVIR”, which represent a drug comprising both components, to facilitate a comprehensive examination of potential synergistic adverse effects (see Figure S1 in Multimedia Appendix 1 for detailed tool settings). For hydroxychloroquine, both “hydroxychloroquine” and its sulfate form, “hydroxychloroquine sulfate”, were applied for analysis (see Figure S2 in Multimedia Appendix 1 for detailed tool settings).

**Figure 2.**
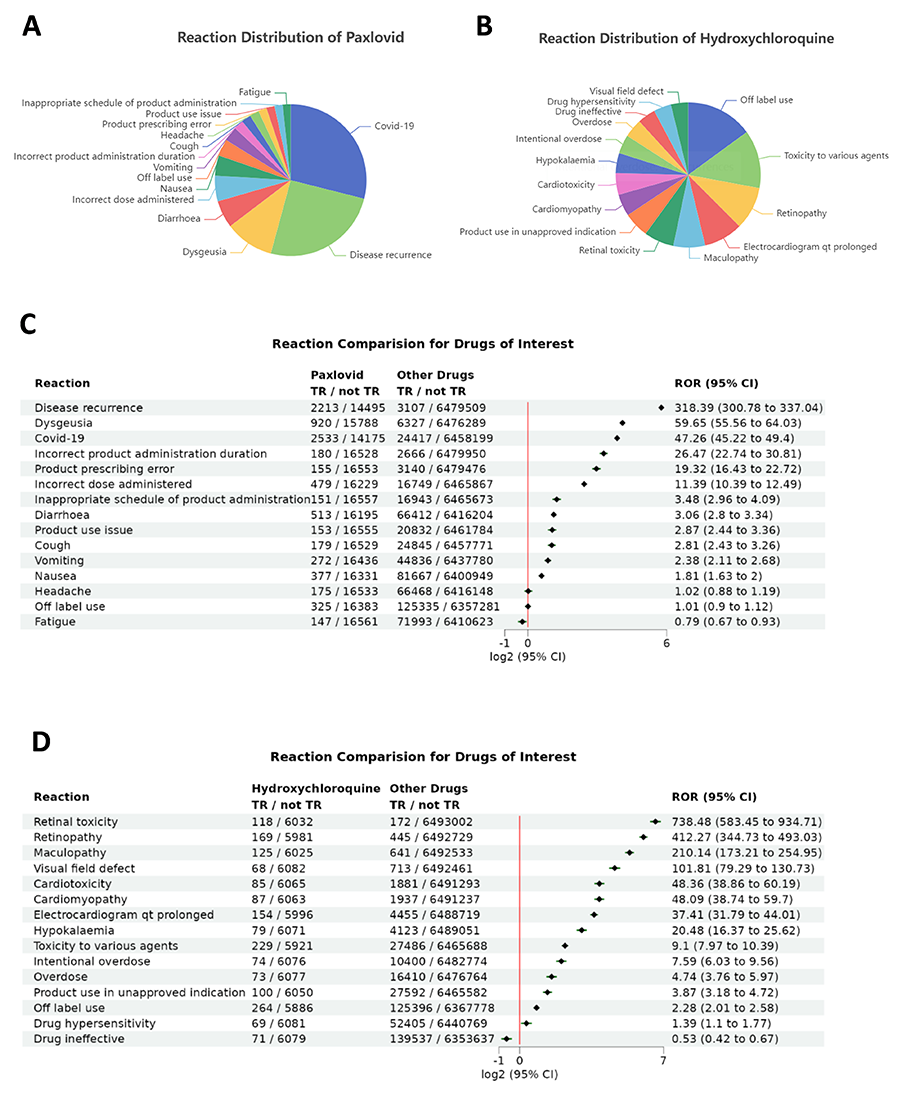
Most relevant adverse drug reactions (ADRs) associated with Paxlovid and hydroxychloroquine based on FAERS (FDA Adverse Event Reporting System) reports analyzed using the VisDrugs system. Pie charts show the top 15 ADRs reported for Paxlovid (**A**) and hydroxychloroquine (**B**). Forest plots comparing the top 15 ADRs of Paxlovid (**C**) and hydroxychloroquine (**D**) with those of all other drugs. The black diamonds indicate the log2-transformed reporting odds ratios (RORs), and the green lines represent the corresponding log2-transformed 95% CIs. TR: target reaction.

For Paxlovid, from the pie chart and forest plot (Figure 2A and C), we observe that in the top 15 ADRs, 12 ADRs occur more frequently in Paxlovid compared to other drugs. Patients using Paxlovid are more frequently reported to experience disease recurrence, dysgeusia (COVID-19), and taste disorder (Figure 2C). In fact, the presence of COVID-19 is consistent with Paxlovid’s indication, and disease recurrence is a very common phenomenon in COVID-19 cases [27,28]. Dysgeusia and taste disorders are also the most common ADRs associated with COVID-19. Other reported ADRs, such as cough, vomiting, and headache, are also commonly associated with COVID-19, indicating that the ADR reports for Paxlovid can be viewed as part of the disease symptoms to its indication, COVID-19 [29].

This situation in the ADR analysis may occur because health care professionals may report both disease symptoms and drug-related side effects as ADRs when submitting data to FAERS. This highlights the importance of considering the drug’s indication when analyzing ADRs in FAERS. When a drug’s major ADRs align with its intended indication, it might be reasonable to conclude that the drug does not cause additional severe ADRs. Furthermore, the analysis results above are very similar to those obtained by Li et al [29], who used FAERS data to analyze Paxlovid-related ADRs from health care professionals, confirming the feasibility of the analysis method provided by this website.

For hydroxychloroquine, we observe that the most frequently reported ADRs include retinal issues (such as retinopathy, maculopathy, visual field defects, and retinal toxicity), cardiac issues (such as prolonged QT on electrocardiogram and cardiomyopathy), and hypokalemia, which may lead to arrhythmia (Figure 2B). The forest plot also shows that hydroxychloroquine is indeed more likely to cause these ADRs compared to other drugs (Figure 2D).

This finding aligns with existing research, as hydroxychloroquine has been shown in epidemiological studies to pose a potential risk for retinopathy [30,31]. Additionally, cardiac disorders are a significant side effect of hydroxychloroquine. It has been reported that plenty of severely ill COVID-19 patients treated with hydroxychloroquine experienced a corrected QT interval increase [32,33]. In addition, case reports have shown that hydroxychloroquine overdose can lead to hypokalemia [34]. Given that FAERS analysis has highlighted the risk of hypokalemia associated with hydroxychloroquine, this adverse effect warrants closer attention.

Interestingly, hydroxychloroquine was originally an antimalarial drug, but the top ADRs seem not related to malaria, indicating severity of retinal and cardiac issues caused by hydroxychloroquine are of much greater concern to health care providers than any potential ADRs related to malaria itself.

### Reaction Comparison Between Drugs

The “Reaction Comparison Between Drugs” mode allows users to compare the occurrence of specific ADRs between 2 groups of drugs. The following steps are required to perform this analysis: (1) input and select the active ingredient name of the target drug; (2) leave “Control Drugs” as “OTHER DRUGS” to compare the target drug with all other drugs, or delete “OTHER DRUGS” and input the active ingredient name of a specific control drug to compare the ADRs of 2 particular drugs; (3) input and select the ADR names of interest; (4) choose whether to group the analysis by age and gender, or conduct the analysis without grouping; and (5) name both the target drug group and the control drug group, then click “Submit.”

This analysis method allows for a focused comparison of specific ADRs between drugs of the same class, facilitating researchers in identifying safer options within the same drug category.

### Application Example: Comparing ADRs of Hydroxychloroquine and Paxlovid

In this example, the user inputs “hydroxychloroquine” in the target drug field, selecting both “hydroxychloroquine” and “hydroxychloroquine sulfate”. In the control drug field, we use “NIRMATRELVIR/RITONAVIR”. The target reaction field is filled with the most potential ADRs of hydroxychloroquine found in Figure 2, including retinal toxicity (retinopathy, maculopathy, and visual field defect), cardiac ADRs (cardiomyopathy and electrocardiogram QT prolongation), and hypokalemia, which may lead to arrhythmias.

**Figure 3.**
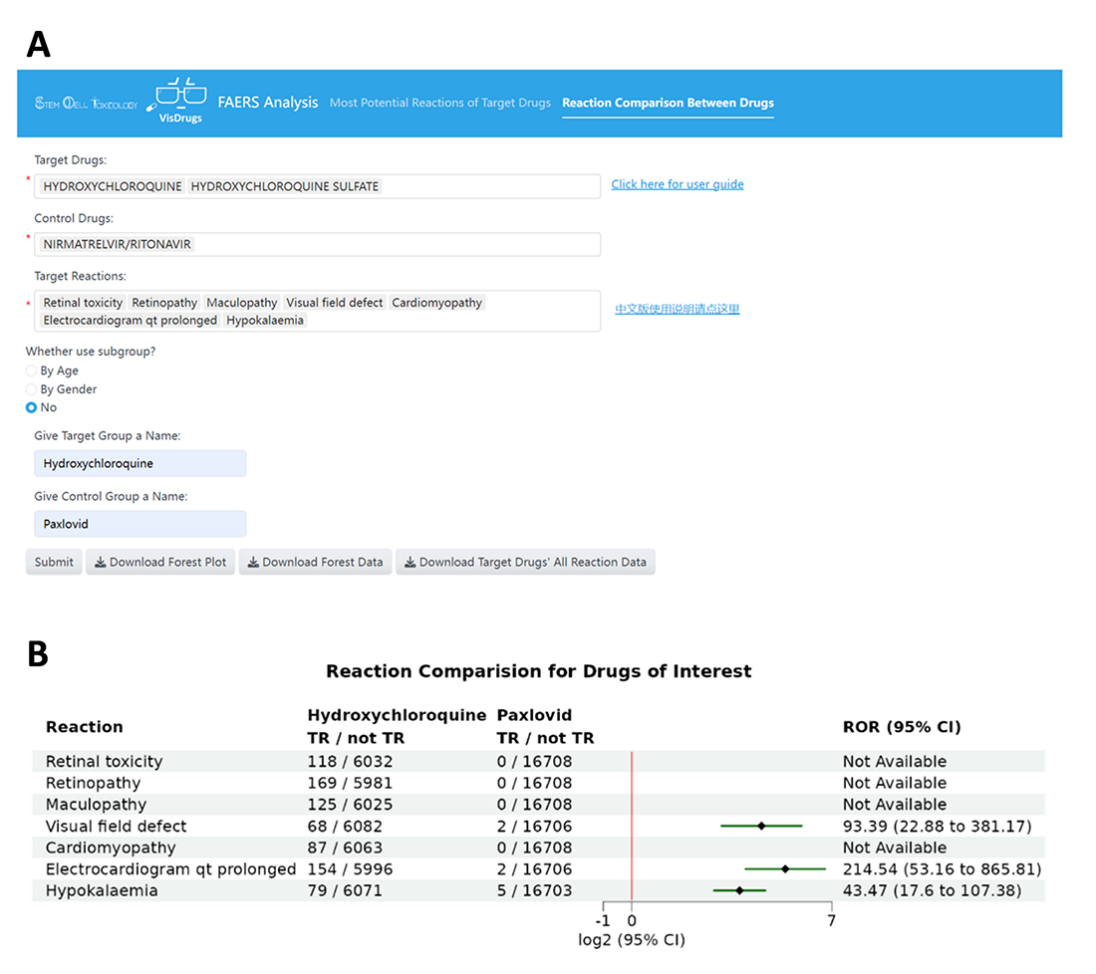
Comparison of retinopathy, cardiomyopathy, and hypokalemia potential of hydroxychloroquine and Paxlovid. (**A**) User input example under the Reaction Comparison Between Drugs model. (**B**) Forest plot comparing the adverse drug reactions (ADRs) of interest between hydroxychloroquine and Paxlovid groups. The black diamonds indicate the log2-transformed reporting odds ratios (RORs), and the green lines represent the corresponding log2-transformed 95% CIs. TR: target reaction.

In the mode without subgroup analysis (Figure 3A), upon submitting the data, the results in the forest plot show that compared to the control drug Paxlovid, hydroxychloroquine indeed tends to cause the aforementioned 7 ADRs (Figure 3B). Conversely, the frequency of these reactions with Paxlovid is extremely low. Among 15,850 cases of Paxlovid use, only a limited number of cases reported related ADRs, suggesting that Paxlovid induces less retinal toxicity, cardiomyopathy, or hypokalemia compared to hydroxychloroquine.

### Application Example: Gender Difference Analysis of Hydroxychloroquine ADRs

In this example, the user inputs “hydroxychloroquine” in the target drug field, selecting both “hydroxychloroquine” and “hydroxychloroquine sulfate”. In the control drug field, “OTHER DRUGS” is retained for comparison with ADRs from all other drugs. The analysis focuses on retinal ADRs (such as retinal toxicity, retinopathy, maculopathy, and visual field defects), cardiac ADRs (including cardiomyopathy and electrocardiogram QT prolongation), and hypokalemia, which may lead to arrhythmias. The data is submitted with a gender-based subgroup analysis.

**Figure 4.**
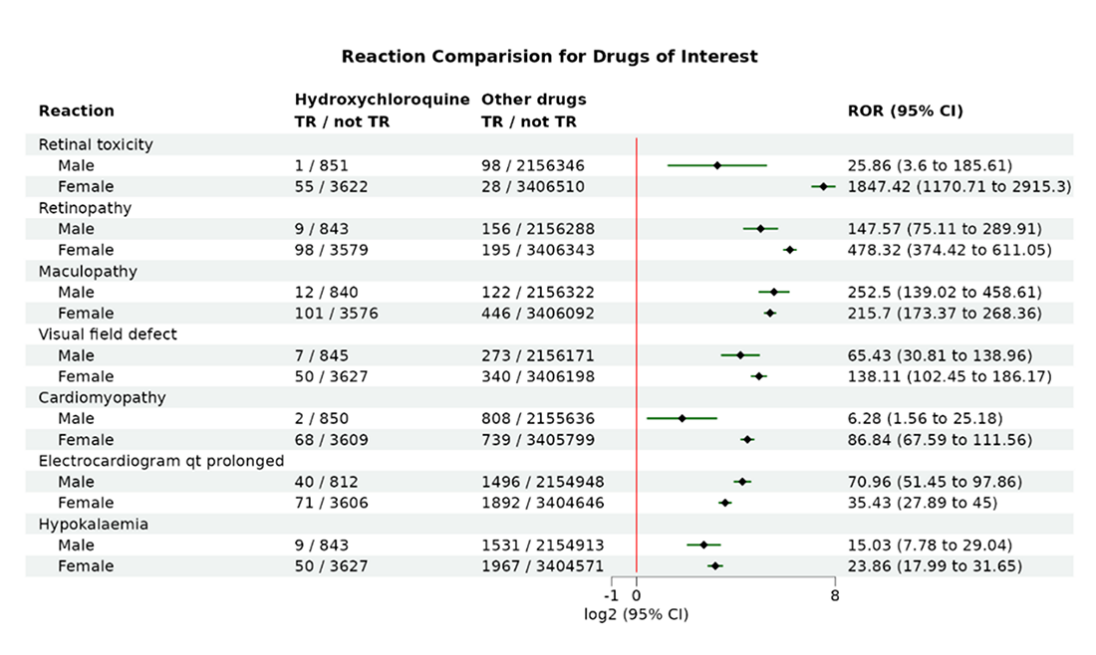
Evaluation of the gender-specific risk of hydroxychloroquine on retinopathy, cardiomyopathy, and hypokalemia. Shown is the forest plot comparing the adverse drug reactions (ADRs) of interest between hydroxychloroquine and other drug groups, subgrouped by gender. The black diamonds indicate the log2-transformed reporting odds ratios (RORs), and the green lines represent the corresponding log2-transformed 95% CIs. TR: target reaction.

The results in Figure 4 indicate that “hydroxychloroquine” is more likely to cause retinal toxicity in female patients, including retinal toxicity (71.44-fold; *z* test *P* value <.001 than in male patients) and retinopathy (3.24-fold; *z* test *P* value =.001 than in male patients). This aligns with an epidemiological study by Jorge et al [35] in 2024, which found that female sex was associated with a higher risk of these adverse effects. Additionally, the results show that “hydroxychloroquine” is more likely to cause cardiomyopathy in female patients (13.82-fold; *z* test *P* value <.001 than male). This finding contrasts with a study by Goldman et al [36] in 2022, which analyzed the FAERS database data from 07/2014 to 09/2019 and did not observe a gender-based difference in cardiomyopathy risk. The discrepancy may be due to their inclusion of reports from non–health care professionals, which could be less accurate. Alternatively, the increase in cardiovascular disease in women during the COVID-19 pandemic, or the possibility that “hydroxychloroquine” may indeed have a greater propensity to cause heart disease in women, could explain this difference. Notably, in the ADRs associated with Paxlovid, we did not observe any cardiac-related ADRs. Additionally, evidence indicates that COVID-19 is more likely to cause viral myocarditis or pericarditis in men [37]. Therefore, we speculate that hydroxychloroquine may have a higher risk of causing heart disease in women, which is a noteworthy finding from this study.

In addition, we conducted a gender-based subgroup analysis of the top 10 most frequently reported ADRs potentially representing ADRs for Paxlovid. The ROR differences between male and female subgroups for these ADRs were all less than 1.5 times (Figure S3 in Multimedia Appendix 1), suggesting that sex differences have minimal impact on the occurrence of these ADRs.

### Application Example: Age Difference Analysis of Hydroxychloroquine ADRs

This example is similar to the previous one, with the main difference being the selection of age-based subgroup analysis. The age cutoff is set at 50 years. Interestingly, when the age is set to 50, the total number of case reports for patients using hydroxychloroquine is nearly equal for those older and younger than 50 years.

**Figure 5.**
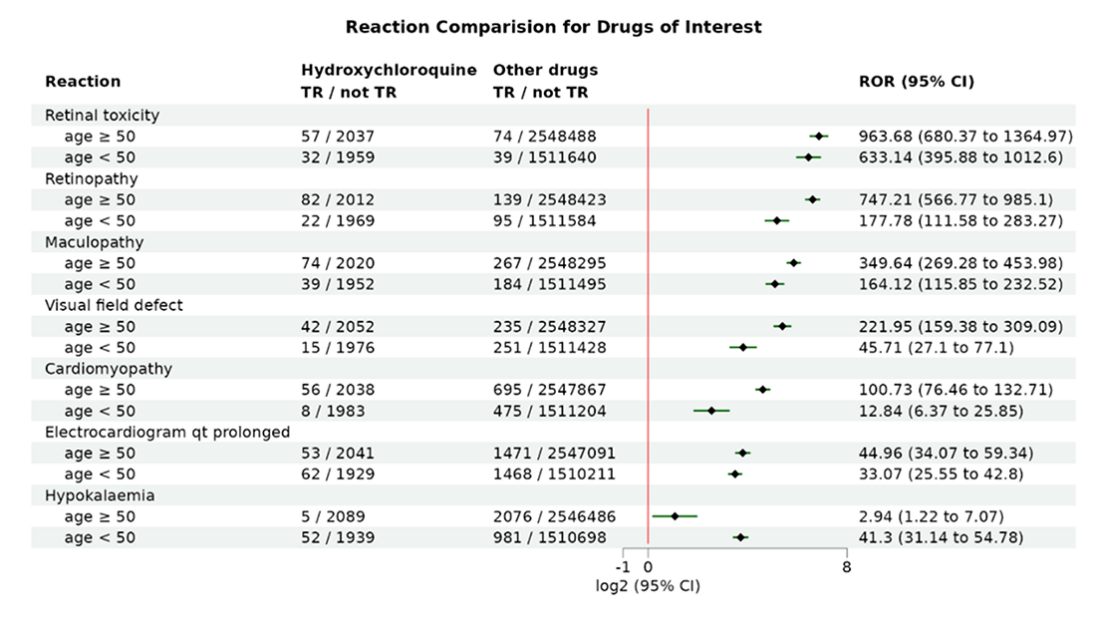
Assessment of the age-specific risk of hydroxychloroquine on retinopathy, cardiomyopathy, and hypokalemia. Shown is the forest plot comparing the adverse drug reactions (ADRs) of interest between hydroxychloroquine and other drug groups, subgrouped by age (age split set to 50 years). The black diamonds indicate the log2-transformed reporting odds ratios (RORs), and the green lines represent the corresponding log2-transformed 95% CIs. TR: target reaction.

From the results (Figure 5), we observe that among the 7 ADRs of interest, only hypokalemia is more likely to occur in younger patients, while other cardiac and retinal ADRs tend to occur more frequently in older patients. Specifically, retinal-related ADRs associated with hydroxychloroquine are more prevalent in the older age group, for example, retinopathy (4.2-fold in older than younger people; *z* test *P* value<.001) and visual field defect (4.86-fold in older than younger people; *z* test *P* value<.001). This trend aligns with the findings in the 2024 study by Jorge et al [35], even though they divided the data at 45 years.

Regarding cardiac issues, while some studies suggest that age is not related to cardiac problems in hydroxychloroquine users [38], analysis of the latest FAERS database indicates that age does indeed influence the occurrence of cardiac-related ADRs, such as cardiomyopathy, with a 7.85-fold higher incidence in older patients compared to younger ones (*z* test *P* value <.001). This suggests that age, a well-established factor in drug-related adverse effects, also plays a significant role in the incidence of cardiac issues with hydroxychloroquine. At the same time, the fact that hydroxychloroquine significantly tends to cause hypokalemia in individuals younger than 50 years also warrants the attention of researchers.

In addition, we conducted an age-based subgroup analysis of the top 10 most frequently reported ADRs potentially representing ADRs for Paxlovid. The ROR differences between ≥50 and <50 year subgroups for these ADRs were all less than 1.5 times (Figure S4 in Multimedia Appendix 1), suggesting that age differences have minimal impact on the occurrence of these ADRs.

### Website Evaluation

To further evaluate the functionality and effectiveness of our website, we used 2 main approaches during the assessment process. First, we conducted internal evaluations through regular group meetings, where students tested the website’s drug ADR analysis functions. They also engaged in discussions to compare the analysis results with real-world reports, refining the analysis process to ensure that the results closely reflected actual ADRs. Second, 13 clinical staff members, including pharmacists, physicians, anesthesiologists, and technicians from institutions such as the Beijing Friendship Hospital, Beijing Tongren Hospital, Peking Union Medical College Hospital, Rizhao Central Hospital, and Rizhao Maternal and Child Health Hospital, participated as volunteers from November 2024 to January 2025 (Table 1) to evaluate the Visdrugs website. With their medical background and extensive clinical experience, they provided invaluable feedback after using the website, contributing to the development of the site and ensuring that it serves its purpose in ADR analysis effectively.

**Table 1.** A list of clinical staff members who participated in evaluating the website.

Office unit	Date of participation	Form of participation	Job title
Department of Pharmacy, Beijing Friendship Hospital, Capital Medical University	November 22, 2024	Website test and online discussion	Licensed pharmacist, senior pharmacist
Department of Otolaryngology, Beijing Friendship Hospital, Capital Medical University	November 22, 2024	Website test and online discussion	Associate chief physician
Department of Neurology, Beijing Tongren Hospital, Capital Medical University	December 19, 2024	Website test and online discussion	Associate chief physician
Department of Neurology, Peking Union Medical College Hospital	December 19, 2024	Website test and online discussion	Associate chief physician
Department of Social Health, Rizhao Maternal and Child Health Hospital, Shandong Province	January 03, 2024	Website test and online discussion	Director of the department, associate chief technician
Department of Blood Transfusion, Rizhao Central Hospital, Shandong Province	January 03, 2024	Website test and online discussion	Director of the department, associate chief technician
Department of Pharmacy, Rizhao Central Hospital, Shandong Province	January 15, 2024	Website test and online discussion	Director of the department, associate chief pharmacist
Department of Pharmacy, Rizhao Central Hospital, Shandong Province	January 15, 2024	Website test and online discussion	Attending pharmacist
Department of Pharmacy, Rizhao Central Hospital, Shandong Province	January 15, 2024	Website test and online discussion	Pharmacist
Department of Pharmacy, Rizhao Central Hospital, Shandong Province	January 15, 2024	Website test and online discussion	Pharmacist
Department of General Surgery, Rizhao Central Hospital, Shandong Province	January 15, 2024	Website test and online discussion	Director of the department, chief physician
Department of Oncology, Rizhao Central Hospital, Shandong Province	January 15, 2024	Website test and online discussion	Director of the department, associate chief physician
Department of Anesthesiology, Rizhao Central Hospital, Shandong Province	January 15, 2024	Website test and online discussion	Deputy director of the department, associate chief anesthesiologist
Department of Internal Medicine, Urumqi Traditional Chinese Medicine Hospital	January 16, 2025	Website test and online discussion	Attending doctor
Department of Reproduction, First Affiliated Hospital of Xinjiang Medical University	January 20, 2025	Website test and online discussion	Attending doctor
Department of Hepatology, Autonomous Region People’s Hospital	20 January, 2025	Website test and online discussion	Attending doctor

## Discussion

### Overview

The FDA FAERS serves as a valuable resource for identifying potential safety concerns associated with drugs and has been widely used in various studies to monitor and analyze drug-related adverse effects [4,5,20,39]. Despite the wealth of data available in FAERS, its vast size makes data utilization heavily reliant on bioinformatics technologies. While platforms like VigiAccess and OpenVigil FDA have made significant strides in simplifying the data retrieval process [16,18], they still present notable limitations in analytical flexibility and usability for non-expert users. VigiAccess allows users to explore ADR reports for individual drugs with summary statistics such as geographical distribution, gender, and age, but it lacks functionality for analyzing the influence of demographic factors on specific ADRs or for conducting comparative analyses across multiple drugs. OpenVigil FDA supports disproportionality analysis (eg, ROR, proportional reporting ratio) based on FAERS data and allows basic filtering, yet it does not provide flexible subgroup analyses or direct comparison of ADR profiles between drugs in an integrated, user-friendly environment.

To overcome these limitations, we developed VisDrugs, a web-based platform designed to facilitate comprehensive and intuitive ADR analysis for clinicians and researchers. VisDrugs uses an automated, standardized data processing workflow to clean and structure FAERS data using consistent nomenclatures (MedDRA PTs for ADRs and active substance names for drugs). Beyond basic retrieval and disproportionality analysis, VisDrugs offers unique capabilities, including flexible subgroup analysis by gender and age, as well as side-by-side comparison of ADR profiles across 2 drugs—features not available in existing public platforms. These advanced yet accessible functionalities aim to empower health care professionals and researchers to perform more nuanced and clinically relevant evaluations of drug safety without requiring extensive bioinformatics expertise.

From the examples provided in this study, we can see that the ADRs for COVID-19 drug Paxlovid and anti-malarial drug hydroxychloroquine obtained from the VisDrugs platform are highly consistent with those reported in previous studies. For Paxlovid, we primarily observed ADEs related to its use in treating COVID-19, which highlights the importance of distinguishing between ADRs related to the drug itself and those associated with the disease being treated. For hydroxychloroquine, the platform not only confirmed the commonly reported retinal and cardiac adverse effects, but through subgroup analysis, we also identified that retinotoxicity, retinopathy, and cardiomyopathy tend to occur more frequently in females and in individuals aged 50 years or older. These findings align with results from epidemiological studies and previous FAERS analyses [30-33,35,38], demonstrating the accuracy and practical utility of the platform’s ADR analysis functionality.

Additionally, when analyzing the ADRs of an effective drug like Paxlovid [40], the top ADRs often include many reactions related to its intended indication, and the ROR confirms that these indication-related ADRs are indeed associated with Paxlovid usage. In contrast, when analyzing a drug with strong side effects, such as hydroxychloroquine [41], many of the top ADRs are non-indication related, and ROR shows that these non-indication ADRs are associated with hydroxychloroquine use. This suggests that the number of indication-related versus non-indication-related ADRs in the top drug usage-relevant ADRs, along with the severity comparison between them, may help evaluate the suitability of a drug. It can be inferred that when the severity of a drug’s side effects does not exceed the severity of its indication, the indication-related ADRs are more likely to catch the attention of the reporters, and thus, most ADRs will be related to the drug’s intended use. However, if a drug’s side effects are so severe that they overshadow the indication, reporters are more likely to focus on reporting these non-indication-related ADRs. For example, in the case of hydroxychloroquine, where the ADR analysis reveals a lack of indication-related ADRs and the drug’s adverse effects lead to severe outcomes, this may indicate that the drug is not suitable for treating that disease.

### Limitations and Future Perspectives

While the development of the VisDrugs platform fills a gap in the existing FAERS analysis tools by offering 1-click visualization of ADR data, it does have some limitations. First, regarding the data source, VisDrugs relies primarily on FAERS data, which are collected through voluntary reports from health care professionals and others. Voluntary reporting bias is a well-known limitation of spontaneous reporting systems such as FAERS. Underreporting is common, particularly for known or less severe ADRs, whereas overreporting may occur following media attention or regulatory actions. Additionally, reporting patterns differ between health care professionals and patients. While health care professional reports are generally more clinically detailed and accurate, patient reports may be broader but less specific. Although VisDrugs enhances data reliability by focusing exclusively on reports submitted by health care professionals, addressing the impact of underreporting and overreporting remains an important challenge for future work. From an analytical perspective, the platform currently provides ADR frequency pie charts and ROR forest plots for analyzing 1 or 2 drugs. While these are useful, more advanced visualization functions are still in development. The platform continues to need optimization and upgrading based on user feedback. Moreover, the current version does not integrate in-depth biological information, such as molecular mechanisms or drug-target interactions, which would further enhance the platform’s utility and depth of analysis.

Additionally, this study did not include a formal usability or performance evaluation of the VisDrugs platform. Structured evaluation studies (eg, user usability testing, performance benchmarking, and accuracy validation) are planned as part of future work to further validate and refine the system.

Looking ahead, we plan to expand the capabilities of the VisDrugs platform by adding more advanced visualization features, offering users the ability to specify the year for analysis, and enabling subgroup analysis based on factors like geographical location. We also aim to incorporate information on known drug indications, molecular targets, and links to ADR annotations to improve the platform’s readability and usability. Furthermore, we envision integrating artificial intelligence technologies, such as machine learning models, to assist in ADR calculation or to use predictive models to interpret user-generated visualized results. These improvements will enhance the efficiency of drug safety evaluations.

### Conclusions

Visdrugs offers a highly streamlined and user-friendly platform for analyzing ADRs, bridging the gap between raw FAERS data and actionable insights into drug safety. What this website focuses on is transforming raw data into accessible and systematic knowledge by establishing a comprehensive analytical platform. This enables medical researchers to efficiently access post-marketing drug safety information without spending time on bioinformatics analyses or coding, allowing them to focus on evaluating the ADR profiles of different drugs. By doing so, VisDrugs contributes to advancing the assessment and monitoring of post-marketing drug safety.

Using the computational methods integrated into this platform, our previous study has already evaluated the pregnancy safety profiles of 2 hepatitis B antiviral drugs, adefovir and entecavir [39]. Since then, we have further enhanced the platform’s capabilities. Now, it not only supports the analysis of the most common ADRs of individual drugs and the comparison of the toxicity profiles of 2 drug groups, but also enables subgroup analyses based on sex and age. This allows users to investigate the influence of demographic factors on drug safety, providing deeper insights into the differential effects of ADRs.

As demonstrated in our examples, VisDrugs significantly accelerates the process of comprehensive ADR evaluation. In just minutes, users can perform an in-depth assessment of a drug’s safety profile, uncover patterns of ADRs across different demographic groups, and explore the impact of specific factors such as age and sex. This remarkable efficiency makes VisDrugs a powerful tool for advancing drug safety evaluation, improving our ability to monitor, understand, and mitigate ADRs in diverse populations.

## Supplementary material

10.2196/71519 Multimedia Appendix 1 Supplementary figures (Figures S1-S4).

10.2196/71519 Checklist 1 Software/Tools Papers checklist.

## Abbreviations

ADR: adverse drug reaction
ASCII: American Standard Code for Information Interchange
FAERS: US Food and Drug Administration Adverse Event Reporting System
FDA: US Food and Drug Administration
MedDRA: Medical Dictionary for Regulatory Activities
PT: preferred term
ROR: reporting odds ratio
