# Supplementary material for: Visualization of FDA Adverse Drug Reaction Reports: Development and Usability Study of the VisDrugs Web Server
Source: JMIR Form Res. 2025 Jul 31;9:e71519. doi: 10.2196/71519 (PMC12312990; doi:10.2196/71519)
Supplement: Multimedia Appendix 1 [file formative-v9-e71519-s001.docx]

Visualization of FDA Adverse Drug Reaction Reports: Development and Usability Study of the VisDrugs Web Server

Renjun Yang^a,b^, Nuoya Yin^a,b^, Yang Zhang^c^, Francesco Faiola^a,b,^*.

^a^State Key Laboratory of Environmental Chemistry and Ecotoxicology, Research Center for Eco-Environmental Sciences, Chinese Academy of Sciences, Beijing 100085, China

^b^College of Resources and Environment, University of Chinese Academy of Sciences, Beijing 100049, China

^c^Department of Pharmacy, Beijing Friendship Hospital, Capital Medical University, Beijing 100050, China

^*^Correspondence: Dr. Francesco Faiola, Research Center for Eco-Environmental Sciences, Chinese Academy of Sciences, 18 Shuangqing Road, Beijing 100085, China; Telephone/Fax: +8601062917609; Email: faiola@rcees.ac.cn.


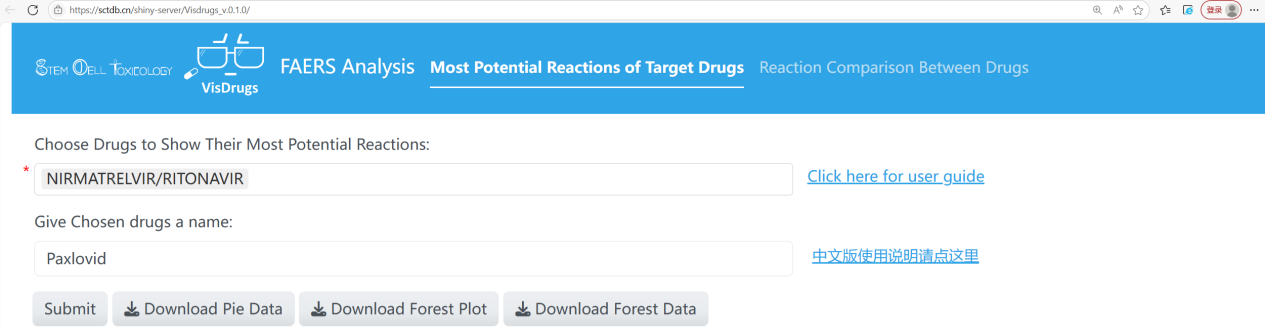


**Figure S1. User settings for analysis of Paxlovids ADRs**


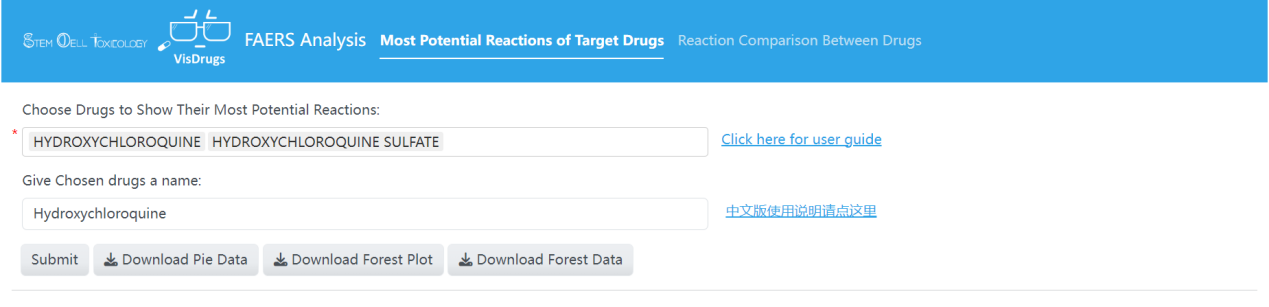


**Figure S2. User settings for analysis of Hydroxychloroquine ADRs**


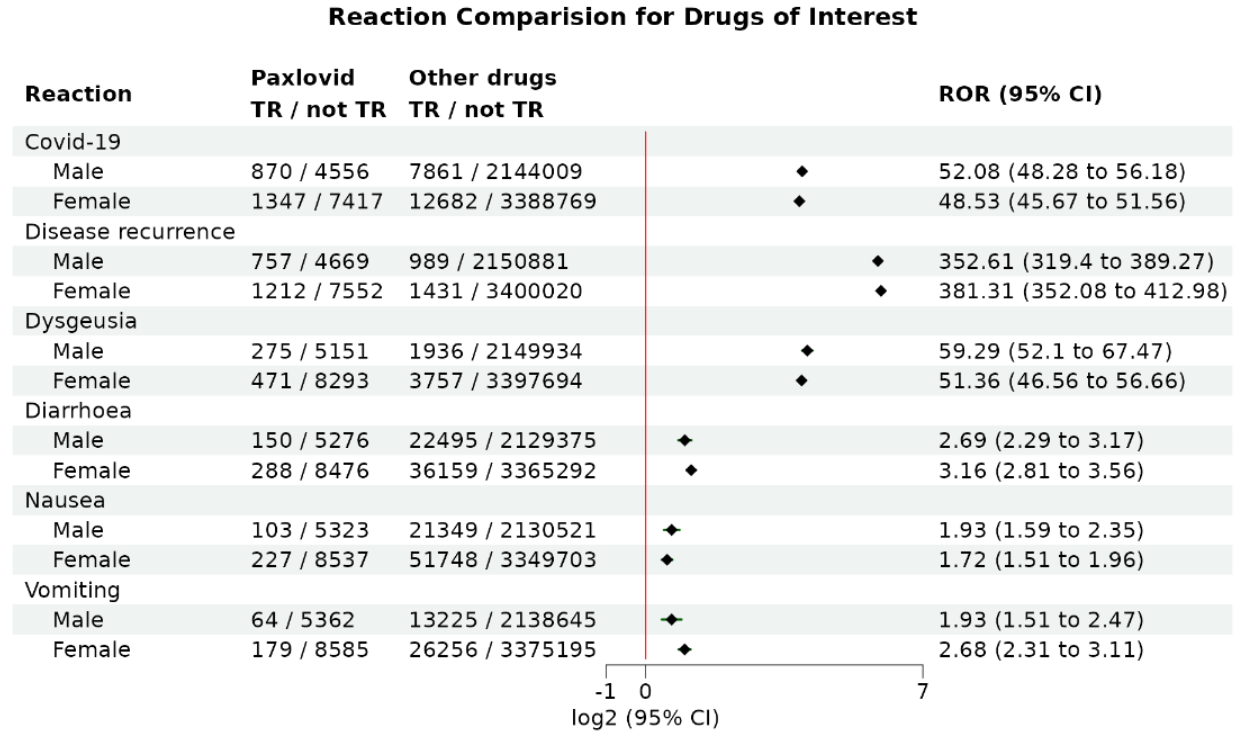


**Figure S3. Assessment of the gender-specific risk of Paxlovid.** Shown is the forest plot comparing the PTs of interest between Hydroxychloroquine and other drugs groups, subgrouped by gender. The black diamonds indicate the log2-transformed Reporting Odds Ratios (RORs), and the green lines represent the corresponding log2-transformed 95% confidence intervals.


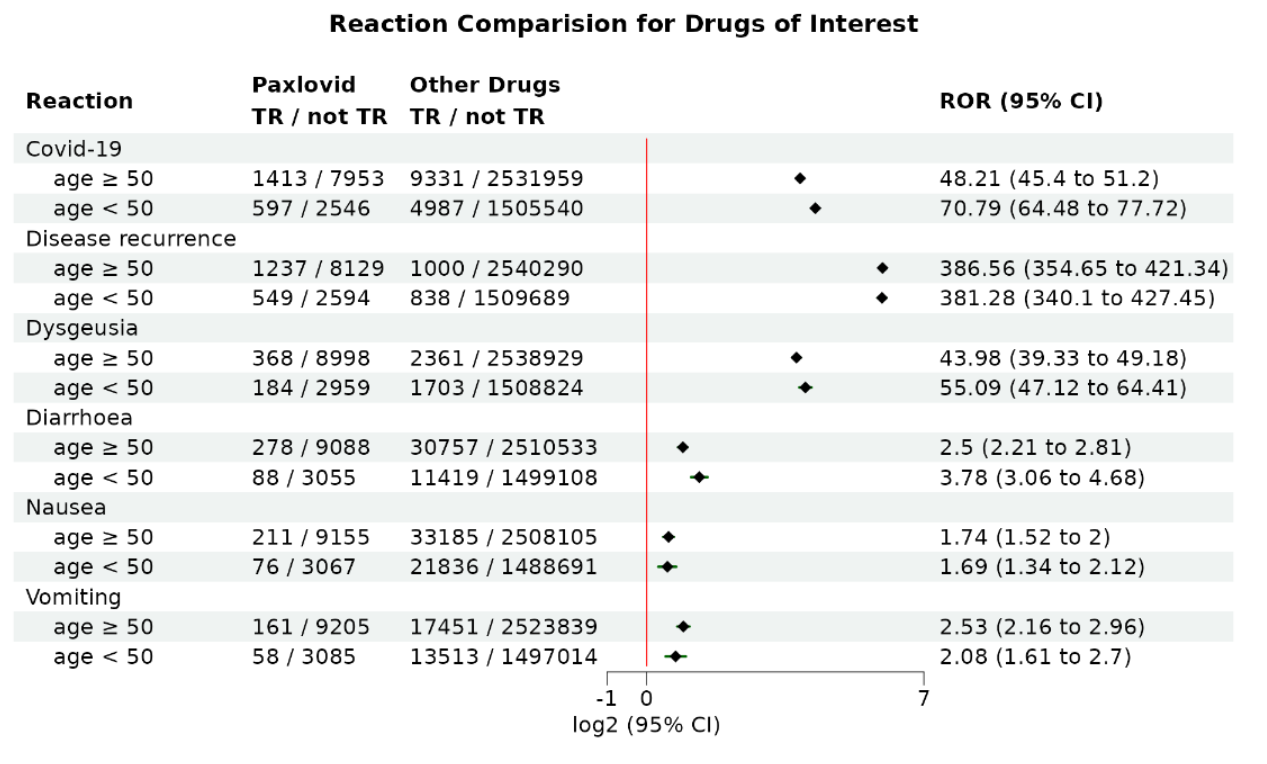


**Figure S4. Assessment of the age-specific risk of Paxlovid.** Shown is the forest plot comparing the PTs of interest between Paxlovid and other drugs groups, subgrouped by age (age split set to 50 years). The black diamonds indicate the log2-transformed RORs, and the green lines represent the corresponding log2-transformed 95% confidence intervals.
