# Supplementary material for: Visualization of FDA Adverse Drug Reaction Reports: Development and Usability Study of the VisDrugs Web Server
Source: JMIR Form Res. 2025 Jul 31;9:e71519. doi: 10.2196/71519 (PMC12312990; doi:10.2196/71519)
Supplement: Checklist 1 [file formative-v9-e71519-s002.docx]

# Software/Tools Papers Checklist

**Manuscript Title:** Visualization of FDA Adverse Drug Reaction Reports: Development and Usability Study of the VisDrugs Web Server
**Authors:** Renjun Yang, Nuoya Yin, Yang Zhang, Francesco Faiola*.
**Journal:** JMIR Formative Research

**1. Background and Objective**

**The paper should clearly state the background, problem addressed, and objectives of the software/tool.**

We discussed this part of content mainly in introduction part. The manuscript provides a detailed background on the challenges of adverse drug reaction (ADR) data analysis using FAERS data, and highlights the need for user-friendly, interactive, and interpretable web-based tools. The objective of Visdrugs is to provide a publicly accessible platform that allows clinicians, researchers, and regulatory bodies to analyze ADR signals without requiring advanced programming skills.

### 2. Description of the Software/Tool

**The manuscript should describe the software/tool, including technical specifications, platforms supported, and unique features.**

In the Methods section, we have provided detailed descriptions in the following subsections:

Website framework:

Describes the server configuration and underlying architecture of the VisDrugs platform.

Data retrieving, preprocessing, and visualization:

Details the data sources, preprocessing pipeline, and visualization workflow implemented in the platform.

Calculation of Reporting Odds Ratio (ROR) and Statistical Significance Analysis:

Explains the methodology for ROR calculation, as well as the procedures and thresholds used for statistical significance testing.

### 3. Availability

**The manuscript should state whether the software/tool is freely available, provide URLs, and licensing information.**

The manuscript contains an availability and requirements section as follows:

Project name: Visdrugs 1.0.

Interactive website available at: http://sctdb.cn/shiny-server/Visdrugs_v.0.1.0/

Step-by-step user guide available at: "Click here for user guide" link on the VisDrugs web interface or https://sctdb.cn/shiny-server/Visdrugs_v.0.1.0/INFO/INFO_EN.htm.

Source code available at: https://github.com/mrpotatod/Visdrugs_v.0.1.0.

Operating Systems: Linux, Windows.

License: Apache License 2.0

### 4. Evaluation/Validation

**The manuscript should report any evaluation, validation, or user testing of the tool.**

The tool has been internally validated using multiple historical FAERS datasets to ensure consistent and accurate ADR signal visualization and calculation. We also conducted usability testing with a group of 16 clinical researchers and pharmacovigilance experts. Feedback from users indicated that Visdrugs greatly lowers the technical barrier for FAERS data exploration and enhances interpretability.

### 5. Limitations

**The manuscript should discuss limitations of the software/tool and possible future improvements.**

The manuscript provided a comprehensive Limitations section. We explicitly discuss the inherent limitations of FAERS data, which used by Visdrugs include voluntary reporting bias and differences between healthcare professional and patient reports. We also acknowledge current limitations of Visdrugs in visualization capabilities and the lack of integrated biological information. Furthermore, we highlight that formal usability and performance evaluations have not yet been conducted for Visdrugs, and we outline concrete plans for future enhancements to address these limitations.

### 6. Conclusions

**The manuscript should provide a clear conclusion summarizing the contribution of the software/tool.**

**We have provided a clear conclusion in the revised Discussion section. The conclusion summarizes that VisDrugs offers a highly streamlined and user-friendly platform for analyzing ADRs, bridging the gap between raw FAERS data and actionable drug safety insights. By transforming complex FAERS data into accessible, systematic knowledge, VisDrugs empowers clinicians and researchers—without requiring bioinformatics expertise—to efficiently evaluate post-marketing drug safety profiles, perform subgroup analyses, and investigate demographic influences on ADRs. This contributes to advancing the assessment, monitoring, and mitigation of adverse drug reactions across diverse populations.**

### 7. Funding

**The paper should report funding sources that supported development of the software/tool.**

The funding sources are listed in Acknowledgements as follows:
This work was supported by the Strategic Priority Research Program of the Chinese Academy of Sciences [grant number XDB0750300], the National Natural Science Foundation of China [grant numbers 22021003, 22476209, 22193052], the Science and Technology Program of Tongzhou District of Beijing [WS2025054], Beijing Municipal Natural Science Foundation [funding number IS23120], and Beijing Overseas Talents Center High-level Foreign Talent Project [funding number C2022001].

### 8. Conflict of Interest

**The paper should disclose any potential conflicts of interest.**

The authors declare no conflicts of interest related to this work.
